# Comparison of pleural effusion features and biomarkers between talaromycosis and tuberculosis in non-human immunodeficiency virus-infected patients

**DOI:** 10.1186/s12879-019-4376-6

**Published:** 2019-08-27

**Authors:** Ye Qiu, Wen Zeng, Hui Zhang, Xiaoning Zhong, Shudan Tang, Jianquan Zhang

**Affiliations:** Department of Respiratory Critical Care Medicine, The First Affiliated Hospital of Guangxi Medical University, Nanning, 530021 Guangxi China

**Keywords:** Talaromycosis, Pleural effusion, Biomarkers, Tuberculosis

## Abstract

**Background:**

Due to the similar clinical, lung imaging, and pathological characteristics, talaromycosis is most commonly misdiagnosed as tuberculosis. This study aimed to identify the characteristics of talaromycosis pleural effusion (TMPE) and to distinguish TMPE from tuberculosis pleural effusion (TPE).

**Methods:**

We enrolled 19 cases each of TMPE and TPE from Guangxi, China. Patients’ clinical records, pleural effusion tests, biomarker test results, and receiver operating characteristic curves were analyzed.

**Results:**

In total, 39.8% (65/163) of patients exhibited serous effusion, of whom 61 were non-human immunodeficiency virus (HIV)-infected patients; 68.85% of the non-HIV-infected patients (42/61) had TMPE. Thoracentesis was performed only in 19 patients, all of whom were misdiagnosed with tuberculosis and received long-term anti-tuberculosis treatment. In four of these patients, interleukin (IL)-23, IL-27, and interferon-gamma (IFN-γ) measurements were not performed since pleural effusion samples could not be collected because the effusion had been drained prior to the study. In the remaining 15 patients, pleural effusion samples were collected. *Talaromyces marneffei* was isolated from the pleural effusion and pleural nodules. Most TMPEs were characterized by yellowish fluid, with marked elevation of protein content and nucleated cell counts. However, neutrophils were predominantly found in TMPEs, and lymphocytes were predominantly found in TPEs (both *p* < 0.05). Adenosine deaminase (ADA) and IFN-γ levels in TMPEs were significantly lower than those in TPEs (all *p* < 0.05) and provided similar accuracies for distinguishing TMPEs from TPEs. IL-23 concentration in TMPEs was significantly higher than that in TPEs (*p* < 0.05), and it provided similar accuracy for diagnosing TMPEs. IL-27 concentrations in TMPEs were significantly lower than those in TPEs (all *p* < 0.05) but was not useful for distinguishing TMPE from TPE.

**Conclusions:**

Talaromycosis can infringe on the pleural cavity via the translocation of *T. marneffei* into the pleural space. Nonetheless, this phenomenon is still commonly neglected by clinicians. TMPE is a yellowish fluid with exudative PEs and predominant neutrophils. Higher neutrophil counts and IL-23 may suggest talaromycosis. Higher lymphocyte counts, ADA activity, and IFN-γ concentration may suggest tuberculosis.

**Electronic supplementary material:**

The online version of this article (10.1186/s12879-019-4376-6) contains supplementary material, which is available to authorized users.

## Background

The dimorphic fungus *Talaromyces* (previously *Penicillium*) *marneffei* causes a life-threatening mycosis, talaromycosis (previously penicilliosis), in immunocompromised persons living in or traveling from Southeast Asia, China, and India [1, 2]. An increasing number of cases with talaromycosis have been reported among non-human immunodeficiency virus (HIV)-infected patients in recent years [3, 4]. Disseminated talaromycosis in non-HIV-infected patients can infringe on the serous cavity to cause serous effusions, especially pleural effusions (talaromycosis pleural effusions [TMPEs]), which frequently went unrecognized previously [5]. The difficulties and challenges in diagnosing TMPE in non-HIV-infected patients might be related to the rarity of clinical studies regarding TMPE, the non-specificity of its clinical manifestations, low positivity rate of pleural effusion culture in the early stage of the disease, and misdiagnosis as other types of pleural effusion [5, 6]. Thus, guidelines for the diagnosis of pleural effusions caused by talaromycosis have not been established, and the diagnosis of TMPE remains challenging.

Due to the similar clinical signs, lung imaging findings, and pathological examination results, talaromycosis is most commonly misdiagnosed as tuberculosis [5–7]. Furthermore, tuberculosis represents one of the most frequent causes of exudative pleural effusions, with predominant lymphocytes in the pleural fluid [8]. Thus, tuberculosis pleural effusion (TPE) has become the most common misdiagnosis of TMPE. In the present study, we aimed to systematically describe the clinical and laboratory characteristics of TMPE in non-HIV-infected patients. We also determined the level of biomarkers, adenosine deaminase (ADA), Interleukin (IL)-23, IL-27, and interferon-gamma (IFN-γ) in TMPEs and TPEs. Furthermore, we compared the laboratory characteristics and concentrations of these biomarkers between TMPE and TPE. The study’s overall aim was to provide an etiological basis, and to evaluate differential diagnosis value of these biomarkers, for the clinical and differential diagnosis of TMPE and TPE.

## Methods

### Study design, participants, and pleural fluid samples

This study was an ambi-spective cohort study. We screened for talaromycosis in non-HIV-infected patients retrospectively from January 1, 2003 and prospectively from January 1, 2013 to May 31, 2017 at the First Affiliated Hospital of Guangxi Medical University, China, which is a 2750-bed tertiary referral center. Non-HIV-infected patients with TMPE were included in the TMPE group. Between May 31, 2016 and May 31, 2017, after matching based on sex and age, 19 randomly selected non-HIV-infected TPE patients were the control group. For each person in the two groups, patients’ medical records were reviewed retrospectively. Corresponding samples of TMPE and TPE obtained by thoracentesis under sterile conditions, were retrieved from a pleural bank maintained in our laboratory (stored at − 80 °C). Exudates were characterized using Light’s criteria [9, 10].

This study was approved by the Ethical Review Committee of the First Affiliated Hospital of Guangxi Medical University (2018.KY-E-071). The requirement for informed consent was waived for patients in the retrospective part of the study because of the retrospective nature of the study. All adult subjects provided written informed consent, and a parent or guardian of any child participant provided written informed consent on the child’s behalf, in the prospective cohort study according to the protocol, when pleural effusion was initially recognized between May 31, 2016 and May 31, 2017.

At baseline, complete histories were obtained and physical examinations with routine clinical laboratory tests were performed for all patients. Data were recorded on standardized case-report forms. Patients’ clinical records, including basic information (sex and age), medical history (present history, underlying diseases, and previous therapy situations), HIV test result, auxiliary examination results (etiological examination, pathematology, imaging examinations, and pleural effusion tests), and treatments were obtained.

All patients met the following inclusion criteria: (a) The patients were those with definitive causes of talaromycosis or tuberculosis, contributing to their pleural effusions. (b) At the time of the sample collection, none of the patients had received any anticancer treatment, antituberculosis therapy, corticosteroids or other non-steroidal anti-inflammatory drugs. (c) All patients were HIV negative. Pleural effusions were classified into two groups: TPEs and TMPEs.

### Diagnostic criteria for talaromycosis

Talaromycosis were diagnosed based on positive cultures for *T. marneffei,* characterized by dimorphic fungi that grew as a mold at 25 °C, and as yeast at 37 °C from clinical specimens^1–4^ (e.g., pleural effusion, pleura, blood, bone marrow, lymph nodes, sputum, skin scrapings, or bronchoalveolar lavage fluid [BALF]). Alternatively, it was diagnosed when the yeast form of *T. marneffei* was confirmed by cytology and histopathology (from tissues and secretions using Periodic Acid-Schiff [PAS] staining or Wright’s-stain), showing a characteristic morphology that includes a transverse septum [7–10]. Disseminated disease was defined as infection in at least two noncontiguous and sterile sites.

### Definitive diagnosis and inclusion criteria for TMPE

The diagnostic and enrollment for TMPE fulfilled one or more of the following criteria: (a) *T. marneffei* identified in pleural fluid, pleural biopsy, sputum, BALF, or lung tissue using histopathology, cytologic smears, and/or fungal culture [1]; or (b) disseminated talaromycosis was diagnosed after *T. marneffei* was isolated from the other clinical specimens with pleural effusion diagnosed based on 1) the presence on imaging examination of micro-abscesses or/and granulomas on pleural biopsy; 2) clinical presentation typical of talaromycosis showed improved and complete resolution of the effusion after receiving antifungal treatment alone, and in exclusion of other diseases (e.g., other bacterial or fungal pulmonary infections, lung cancer, non-infectious interstitial lung disease, heart failure) that cause pleural effusion. Only patients with talaromycosis pleural effusion in non-HIV-infected were included in the TMPE group.

### Definitive diagnosis and inclusion criteria for TPE

Tuberculosis was diagnosed after culture-proven tuberculosis or smear-positive results for acid-fast bacilli and an appropriate response to directed anti-tuberculous therapy. Diagnostic and enrollment for TPEs fulfilled one or more of the following criteria: (a) positive pleural fluid or pleural biopsy or positive sputum Ziehl-Neelsen stain or Lowenstein-Jensen culture, (b) caseous necrotic granulomas on pleural biopsy, or (c) clinical presentation typical of TPE followed by complete resolution of the effusion with only anti-tuberculous treatment [11, 12].

### Exclusion criteria for TMPE and TPE

All patients that met the following criteria were excluded: (a) without a definite diagnosis for talaromycosis or tuberculosis, (b) without pleural effusion, (c) more than one possible etiology based on their effusion, and those with a pleural transudate, hemothorax, or chylothorax were excluded, (d) HIV positive.

### Measurement of ADA, IL-23, IL-27, and IFN-γ

Pleural fluid was collected by diagnostic thoracentesis before the patient had received any treatment; the fluid was rapidly transferred to the laboratory. Pleural fluid and blood sample were centrifuged at 1500 rpm for 10 min at 4 °C, and the supernatants were aliquoted and stored at − 80 °C prior to the measurement of IL-23, IL-27, and IFN-γ. The concentrations of IL-23, IL-27, and IFN-γ in pleural fluid and serum were measured using enzyme-linked immunosorbent assay (ELISA) kits (Cusabio, Wuhan, China) according to the manufacturers’ protocols. The ADA activity in pleural effusion was determined using a colorimetric method (InTec Products, Inc., Xiamen, China) in accordance with the manufacturer’s instructions. The technicians running the assays were blinded to the nature of the samples, and the codes were provided to the statisticians after the construction of the database. Since the pleural effusion of four patients occurred in the retrospective part of this study, we could not obtain their samples because they were drained prior to our study and not stored. Therefore, only 15 TMPE and 19 TPE samples were evaluated in duplicate.

### Determination of HIV status

Two ELISA kits (Enzymun Test, Anti-HIV 1+ 2, Boehringer Mannheim GmbH Diagnostica, Mannheim, Germany) were used to test the sera for the presence of anti-HIV antibodies. Any sera that were negative or positive for anti-HIV antibodies underwent repeat testing at our hospital and at the Guangxi Center for Prevention and Control.

### Statistical analysis

Categorical data are expressed as proportions, and continuous data are expressed as means with standard deviations (SD) for normally distributed variables or as medians (interquartile range, IQR) for non-normally distributed variables. Differences between categorical variables were detected using the chi-squared test. Differences between groups were compared using the Student’s *t*-test, Mann–Whitney U test, one-way analysis of variance (ANOVA), or Wilcoxon rank sum test, as appropriate. Receiver operating characteristic curves were constructed, and areas under the curves (AUCs) were calculated to determine the diagnostic value of each biomarker in the pleural effusion, including sensitivity, specificity, positive likelihood ratio, and negative likelihood ratio [13]. AUCs were compared using the *z* statistic with the Hanley and McNeil procedure. The optimum cut-off values were defined based on their maximum Youden index (sensitivity + specificity − 1). The parameters of diagnostic accuracy are provided along with their respective 95% confidence intervals (CIs) [14]. For all analyses, SPSS version 25.0 (IBM Corp., Chicago, IL, USA) was used, and a two-tailed *p* value < 0.05 was considered significant.

## Results

### Patients’ demographics and pleural samples

**Fig. 1:**
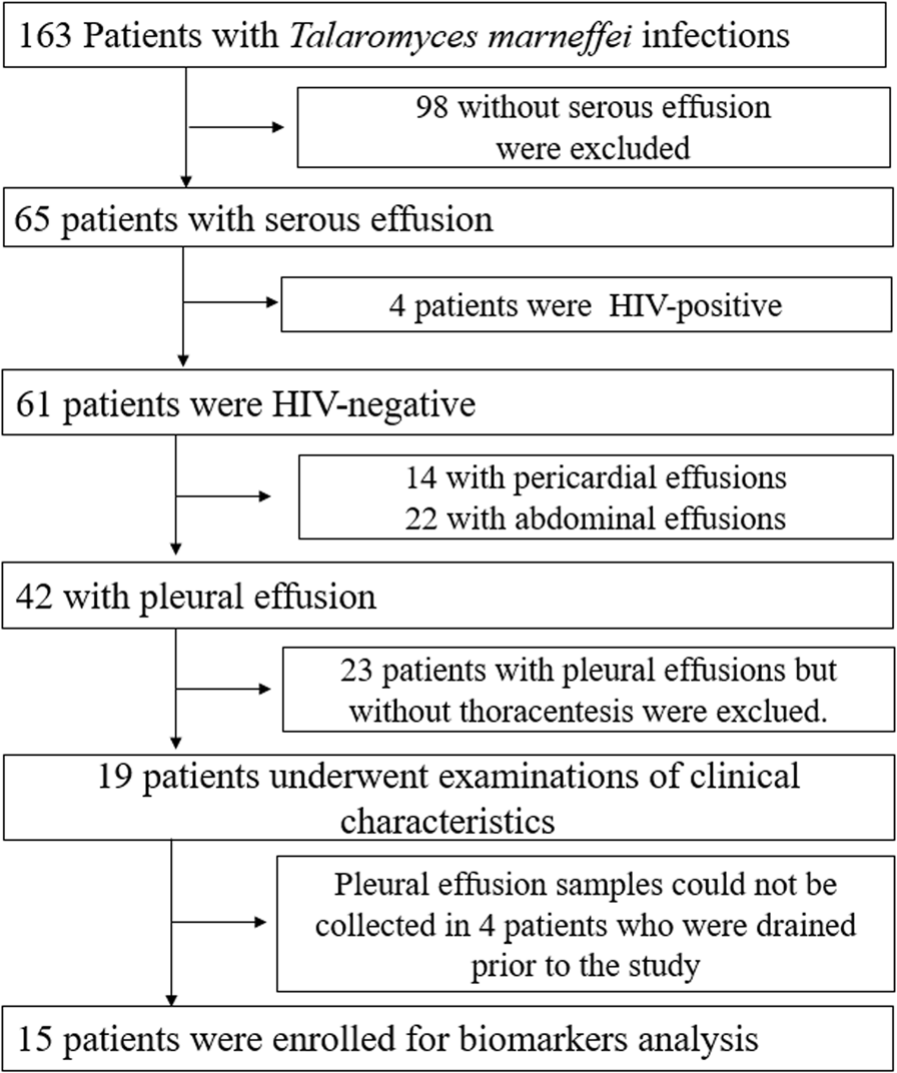
Enrollment, human immunodeficiency virus (HIV) infection condition, and serous effusion

Over the 14-year study period, 163 patients were diagnosed with talaromycosis. Among the 163 patients, 65 (39.88%) exhibited serous effusion, of which, 4 were HIV-positive, and 61 were HIV-negative. Among the non-HIV-infected patients, 14 (22.95%), 22 (36.06%), and 42 (68.85%) exhibited pericardial effusion, abdominal effusion, and pleural effusion, respectively. Among the TMPEs patients, 24 (57.14%) had bilateral and 18 (42.86%) had unilateral pleural effusion. Pleural effusions without an obtainable sample were excluded. Twenty-one patients had moderate or higher pleural effusion; only 19 patients that underwent thoracentesis had the effusate collected. In this study, the participants included 17 adults and 2 children, (age range was 1–68 years). The pleural effusion of four patients could not be obtained because they were drained prior to our study and not stored (Fig. 1).

The 19 non-HIV-infected patients were healthy before the talaromycosis infection and had no pre-existing disease, such as diabetes or connective tissue disease, and had no use of immunosuppressive agents. *T. marneffei*-positive culture results were obtained in tests of the following: sputum (4 cases), bronchoalveolar lavage fluid (3 cases), pleural effusion (2 cases), and pleural nodule (1 case). Additionally, 6 patients were diagnosed with talaromycosis based on histopathology or cytology results of specimens obtained from the pleural nodule (1 case) and lung tissue (5 cases). The five other cases were diagnosed based on the presence of micro-abscesses or granulomas following pleural biopsy and clinical presentation typical of talaromycosis, followed by complete resolution of the effusion with antifungal treatment alone. All patients were misdiagnosed with tuberculosis and had no response to anti-tuberculosis treatment.

All patients presented with fever and cough. Additionally, 14 patients had white sputum, 4 cases had bloody sputum, 14 cases had chest pain, 13 cases had dyspnea, 16 cases had generalized or cervical lymphadenopathy, 10 cases had hepatosplenomegaly, 8 cases exhibited maculopapule, 9 cases had subcutaneous abscess, 4 cases had diarrhea and abdominal pain, while 7 cases had ostealgia.

### Pleural effusion manifestations in TMPE

**Fig. 2:**
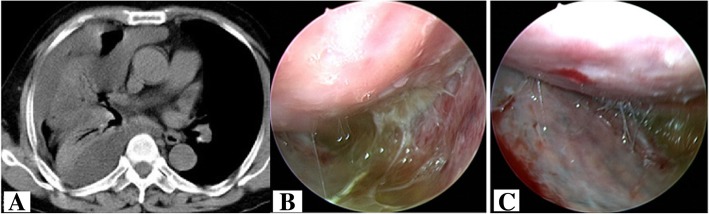
High-resolution computed tomography revealed pleural effusion (**a**). Thoracoscopy showed yellow pleural effusion and adhesions (**b**) and multiple small nodules (**c**) in the pleura

**Fig. 3:**
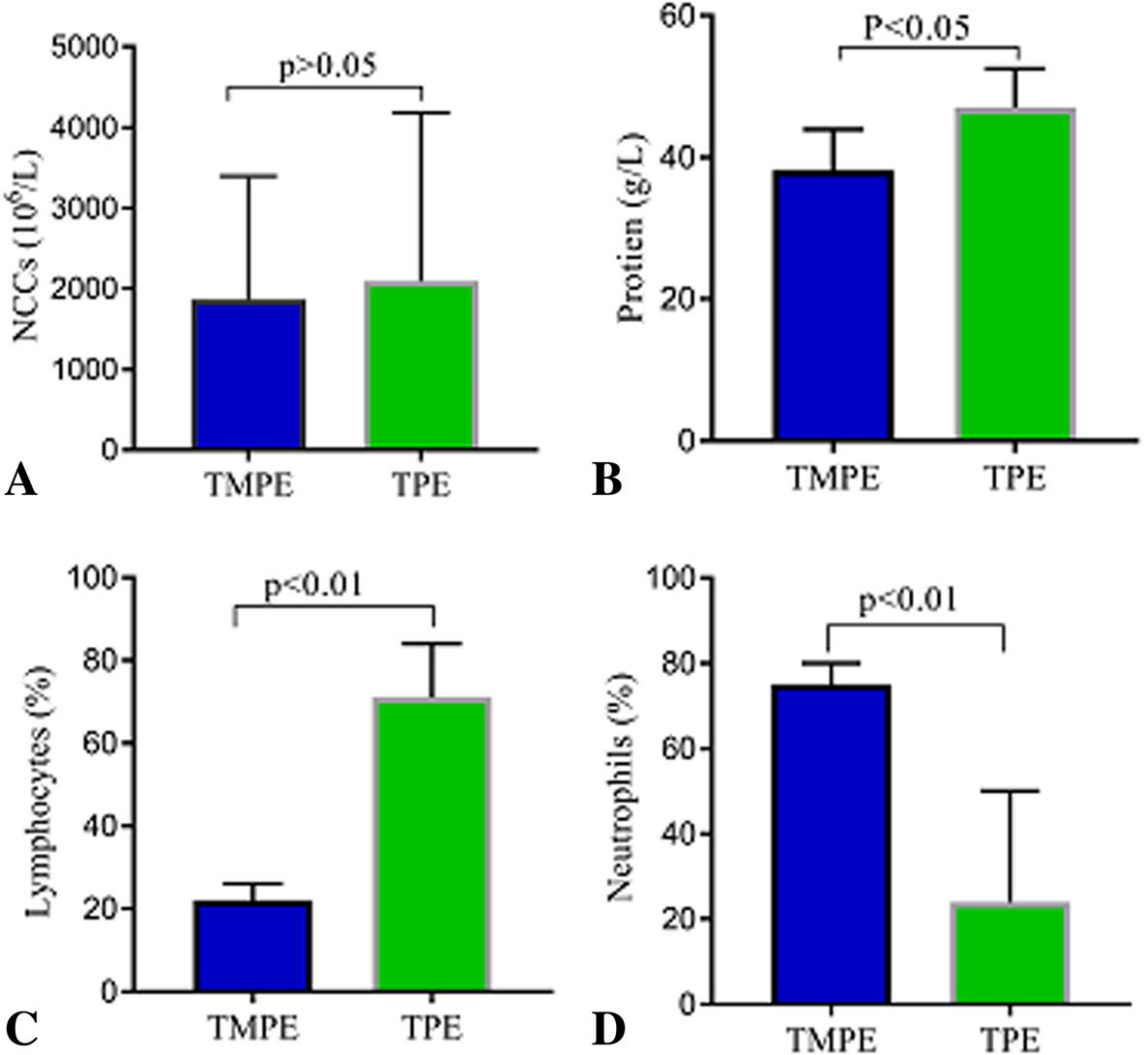
Comparisons of nucleated cell count (NCC) (**a**), protein (**b**), lymphocyte count (**c**), and neutrophil count (**d**) in talaromycosis (*n* = 19) and tuberculous pleural effusion (*n* = 19). *p* < 0.05 compared with tuberculous pleural effusion using analysis of variance followed by Wilcoxon rank sum test

The predominant characteristics of TMPEs included the yellowish color (15/19, 78.9%), hemorrhagic (2/19, 10.5%), and colorless (3/19, 15.8%). Thoracoscopy revealed pleural effusion, fibrous pleural adhesions, and multiple nodules on the pleura (Fig. 2). All TMPE samples showed a positive Rivalta test. Biochemical, cytological, and cytokine-specific characteristics of TPEs and TMPEs are compared in Table 1. Both TPE and TMPE showed a marked elevation of protein content and nucleated cell counts (Fig. 3a and b). However, the protein concentration in TPE was higher than that in TMPE (Fig. 3b). TMPEs and TPEs were all exudative, however, with predominantly neutrophils in TMPEs and predominantly lymphocytes in TPEs. Lymphocyte counts demonstrated markedly higher values in tuberculosis, showing a significant increase in comparison to that in TMPEs (*p* = 0.002) (Fig. 3c). In contrast, neutrophil counts demonstrated markedly higher values in talaromycosis*,* showing a significant increase in comparison to that in TPE (*p* = 0.003) (Fig. 3d).

**Table 1:** Demographic, cytological, and biochemical characteristics of pleural effusions

Variable	TMPE (n = 19)	TPE (n = 19)	p-value
Age (years)	51 (36–58)	55 (25–66)	0.821
Males (n, %)	13, 76.47%	12, 80%	0.742
NCCs (10^6^/L)	1865 (800–2300)	1120 (416–3015)	0.617
Neutrophils (%)	78.0 (71.0–84.0)	20.0 (20.0–39.0)	**0.003** ^**a**^
Lymphocytes (%)	22.0 (15.0–26.0)	71.0 (56.5–77.5)	**0.002** ^**a**^
Protein (g/L)	37.8 (28.0–45.0)	48.0 (40.1–52.6)	**0.035** ^**a**^
LDH (U/L)	278.0 (168.0–532.0)	335.5 (249.0–387.70)	0.341
ADA (U/L)	4.4 (4.0–8.1)	29.9 (13.9–39.4)	**0.002** ^**a**^

Data are presented as median (25th–75th percentile)

^a^ p < 0.05 means statistically significant

### IL-23, IL-27, ADA, and IFN-γ values in TMPEs and TPEs

**Fig. 4:**
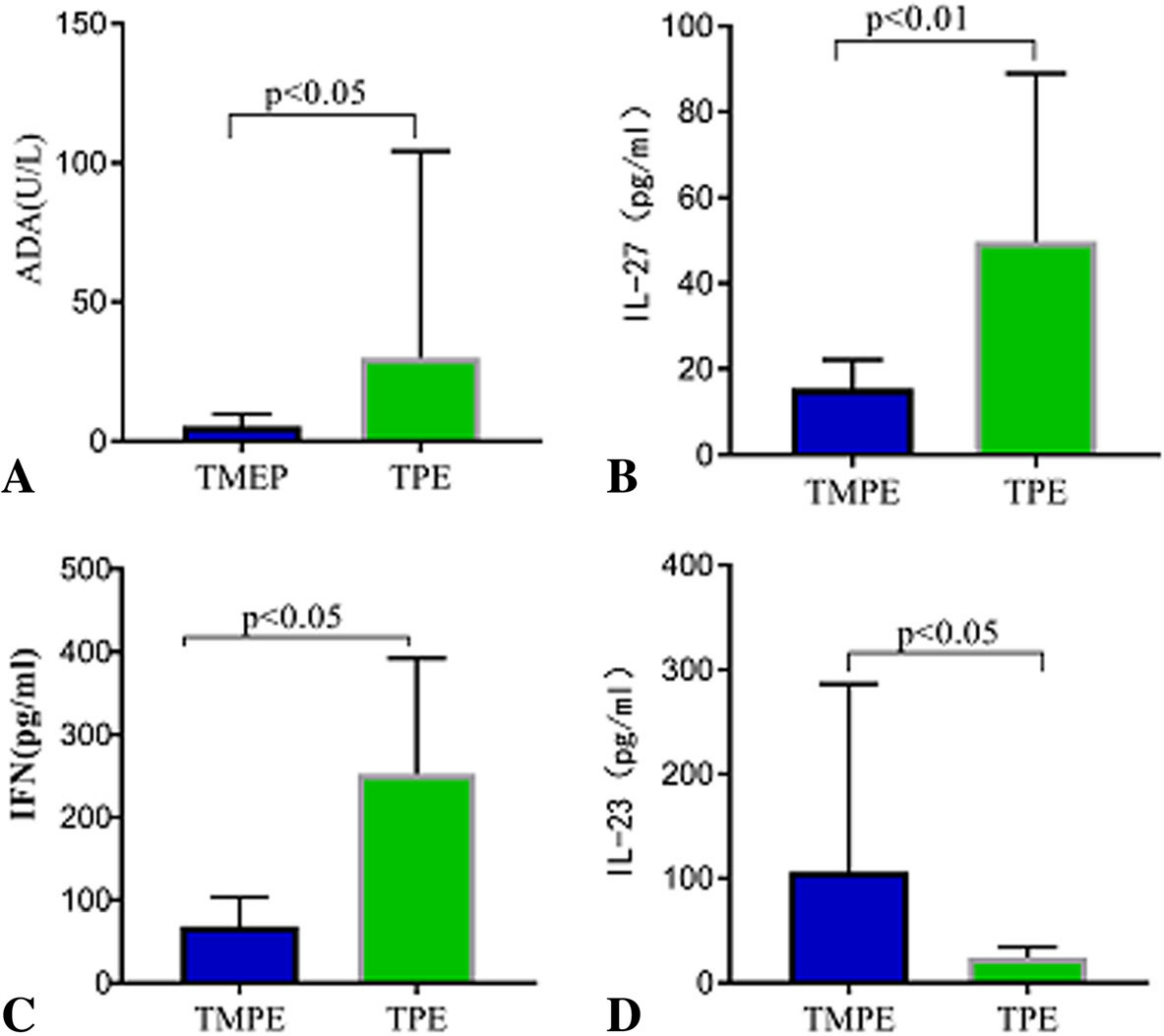
Comparisons of adenosine deaminase (ADA) activity (**a**) in talaromycosis (*n* = 19) and tuberculous pleural effusion (*n* = 19). Comparisons of interleukin (IL)-27 (**b**), interferon (IFN)-γ (**c**), and IL-23 (**b**) in talaromycosis (*n* = 15) and tuberculous pleural effusion (*n* = 19). *p* < 0.05 compared with tuberculous pleural effusion using analysis of variance followed by Wilcoxon rank sum test

In this study, we observed that ADA activity and IL-27 and IFN-γ concentrations in TMPEs were significantly lower than those in TPEs (all *p* < 0.05) (Fig. 4a-c) (Table 2). In contrast, IL-23 concentration in TMPEs was significantly higher than that in TPEs (*p* < 0.05) (Fig. 4d).

**Table 2:** Concentrations of IL-23, IL-27, and IFN-γ in pleural effusion and serum

Variable	TMPE (*n* = 15)	TPE (*n* = 19)	*P*-value
IL-23			
PE (pg/mL)	109.21 (57.74–222.04)	23.5 (11.6–28.8)	**0.026** ^**a**^
Serum (pg/mL)	100.11 (59.81–134.43)	54.79 (34.12–67.26)	**0.024** ^**a**^
IL-27			
PE (pg/mL)	15.44 (11.42–19.12)	49.79 (35.9–81.5)	**0.029** ^**a**^
Serum (pg/mL)	1.49 (0.44–2.37)^a^	1.26 (0.96–2.11)^a^	0.078
IFN-γ			
PE (pg/mL)	68.78 (48.4–89.5)	252.8 (156.5–310.8)	**0.019** ^**a**^
Serum (pg/mL)	6.45 (3.45–7.87)^a^	5.19 (4.30–5.59)^a^	0.458

Data are presented as median (25th–75th percentile)

*ADA* Adenosine deaminase, *IFN* Interferon, *IL* Interleukin, *TMPE* Talaromycosis pleural effusion, *PE* Pleural effusion, *TPE* Tuberculous pleural effusion

^a^ *p* < 0.05 means statistically significant. Compared with the corresponding value using analysis of variance followed by Wilcoxon rank sum test

### IL-23, IL-27, ADA, and IFN-γ values in talaromycosis and tuberculosis serum

In this study, we observed no statistically significant difference in IL-27 and IFN-γ concentrations between talaromycosis and tuberculosis serum. However, IL-23 concentration in talaromycosis serum was significantly higher than that in tuberculosis serum (*p* < 0.05) (Table 2).

### Diagnostic values of IL-23, IL-27, ADA, and IFN-γ in pleural effusions

**Fig. 5:**
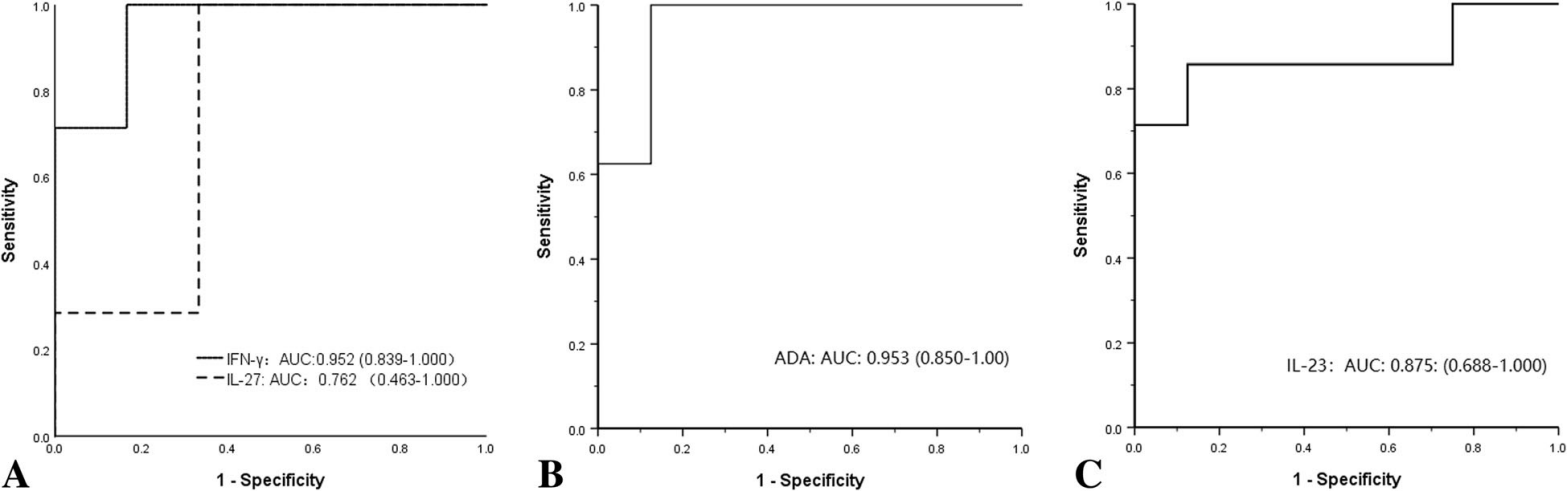
Receiver operating characteristic (ROC) curve analysis of the accuracy of interleukin (IL)-27 and interferon (IFN)-γ concentrations (**a**), adenosine deaminase (ADA) activity (**b**), and IL-23 concentration (**c**) for the diagnosis of talaromycosis pleural effusions

The parameters showing the diagnostic accuracy of IL-23, IL-27, IFN-γ, and ADA are also presented in Fig. 5 and Table 3. The test result was positive to differentiate TMPEs from TPEs with IFN-γ, ADA, and IL-23 having AUCs of 0.952 (95% CI 0.839–1.000) (Fig. 5a); 0.953 (95% CI 0.850–1.00) (Fig. 5d); and 0.875 (95% CI, 0.688–1.000) (Fig. 5c), respectively. Furthermore, IFN-γ, ADA, and IL-23 levels revealed a significant (*p* = 0.006, *p* = 0.002 and *p* = 0.001, respectively) difference between the two diseases. Nevertheless, a comparison of IL-27 ROC curves revealed no significant (*p* = 0.116) differences between the two diseases.

**Table 3:** Receiver operating characteristic curve analyses for IL-23, IL-27, IFN-γ, and ADA in differentiating TMPE from TPE

Biomarker	Optimal cutoff	Sensitivity (%) (95% CI)	Specificity (%) (95% CI)	LR (+)	LR (−)	AUC (95% CI)	*p*-value
IL-23	> 53.07 pg/mL	100 (54.1–100)	88.89 (51.7–99.7)	9	1.12	0.875 (0.688–1.000)	**0.001** ^a^
IL-27	< 35.75 pg/mL	100 (59.0–100)	66.7 (22.3–95.7)	3	0.85	0.762 (0.463–1.000)	0.116
ADA	< 10.9 U/L	100 (63.1–100)	87.5 (47.4–99.7)	8	1.14	0.953 (.0850–1.000)	**0.002** ^a^
IFN-γ	< 129.9 pg/mL	100 (59.0–100)	83.33 (22.3–95.7)	6	1.2	0.952 (0.839–1.000)	**0.006** ^a^

*ADA* Adenosine deaminase, *TMPE Talaromyces marneffei* pleural effusions, *ADA* Adenosine deaminase, *AUC* Area under the curve, *CI* Confidence interval, *IFN* Interferon, *IL* Interleukin, *LR* Likelihood ratio, *TPE* Tuberculous pleural effusion

^a^ *p* < 0.05

### Treatments and outcomes in TMPE

Due to hepatic and renal failure, five patients did not receive antifungal treatment and then died within 2 weeks. Three patients’ conditions worsened despite receiving antifungal therapy, including one patient who experienced cardiac insufficiency and respiratory failure after using itraconazole for 5 days. All three patients developed multiple organ dysfunction syndrome and abandoned their treatment. Subsequently, they were discharged and then died. The other 11 patients who received treatment improved. They continued with treatment using oral antifungal (itraconazole or voriconazole) drugs for time periods ranging from 3 months to 1 year. With effective treatment, pleural effusions typically resolved in 1 week. No patient in the current study used corticosteroids.

## Discussion

*T. marneffei* is a thermally dimorphic pathogenic fungus that causes fatal systemic infection [1]. It can be disseminated to the lung, liver, spleen, skin, central nervous system, blood, and other organs or systems by spreading hematogenously [5, 15]. Previously, TMPE was not described as a common manifestation of *T. marneffei* infections. However, in our study, serous cavity effusion was one of the common presentations in non-HIV-infected talaromycosis patients, especially with pleural effusions. Prior to 2016, there was no evidence of talaromycosis infringing upon the pleural cavity when we reported the first case of *T. marneffei* that was isolated from pleural nodules and pleural effusion based on thoracoscopic pleural biopsy [5]. Nonetheless, this phenomenon is still commonly neglected by clinicians. Prior and current studies that provided direct pathogen-based evidence of talaromycosis fungal translocation into the pleural space were meant to draw the attention of clinicians and imaging physicians. Although pathogen culturing offers 100% diagnostic specificity, it usually takes two or more weeks.

Differential diagnoses of TMPEs include TPEs, malignant pleural effusions, parapneumonic pleural effusion, pleural effusion caused by connective tissue disease, and other viral infections [8–10]. The clinical manifestations (fever, anemia, debilitation, and athrepsia, often accompanied by cough, chest pain, enlarged lymph nodes, and tuberculosis-like pathogenesis); imaging examination findings (patchy infiltrates, extensive consolidation, fibroelastosis, cavity, and pleural effusion); and histopathology (micro-abscess or granulomas) of TMPEs are similar to those of tuberculosis [5, 6, 11]. Additionally, tuberculosis represents one of the most frequent causes of exudative effusions in pleural fluid [11, 16, 17]. Thirdly, the etiological detection rate of tuberculosis in China is very low. The diagnosis of tuberculosis is based, basically, on clinical diagnosis and diagnostic anti-tuberculosis treatment. There is low positivity rate of *T. marneffei* in pleural effusion culture in the early stage of the disease. Furthermore, tuberculosis represents one of the most frequent causes of exudative effusions in pleural fluid. Thus, TPE is the most common clinical misdiagnosis and TMPE is often ignored. Clinicians often misdiagnose TMPE as TPE based on the clinical diagnosis or tend to overlook it while searching for pathogenic evidence. Even with etiological examination, low positivity rate for *T. marneffei* by direct culture for the early detection and histopathology of pleural effusion in the early stages of the disease in HIV-uninfected patients increases the difficulty in diagnosis.

In the present study, all patients were misdiagnosed as having tuberculosis and underwent a long term anti-tuberculosis treatment; however, none of the patients showed any response and the condition was aggravated significantly. After a failed long-term anti-tuberculosis therapy, deterioration of the disease, significant economic burden, and often drug-induced liver and kidney damage due to anti-tuberculosis treatment, resulted in the difficulties in progressing with the next step of treatment. In this study, the mortality rate was 42.10% (8/19). Among these patients, 26.31% (5/19) had drug-induced liver and kidney damage while 15.79% (3/19) had multiple organ dysfunction syndrome. Thus, the misdiagnosis and mistreatment occurring over a long period lead to the deterioration of the condition and poor prognosis. It is therefore very important to determine the etiology and accurate diagnosis in a timely manner. Repeated pleural effusion culture and biopsy using a thoracoscope are helpful for diagnosis. However, it is an invasive test and requires expertise and time (several weeks). Thus, the analysis of the pleural fluid clinically and the biomarkers may provide important information with respect to the diagnosis and differential diagnosis. In this context, we analyzed the clinical and biological markers of pleural effusions caused by talaromycosis and compared them with those of tuberculosis. The current findings may allow for a safer, more rapid, and accurate auxiliary diagnostic method.

In this study, TMPEs were primarily yellowish, and both bilateral and unilateral effusions were observed. Laboratory findings showed that all cases of TMPEs were exudative, with predominant neutrophils; this suggests that TMPE is characterized by a purulent infection. In contrast, TPEs comprised of lymphocytes predominantly. Moreover, the IL-23 concentration was significantly higher in TMPEs, whereas ADA activity, IL-27, and IFN-γ concentration were significantly lower in TMPEs.

ADA is primarily a T lymphocyte enzyme, but the activity varies based on the level of cellular differentiation. ADA activity is higher in lymphocytic pleural effusions of tuberculous origin [17, 18]. IFN-γ is traditionally regarded as a proinflammatory factor and as the signature cytokine of T helper 1 (Th1)-dominated autoimmune processes. IFN-γ plays a key role in macrophage activation, inflammation, host defense against intracellular pathogens, Th1 cell responses, tumor surveillance, and immunoediting. Furthermore, IFN-γ associates with Th1-driven immune responses, not only in the effector phase, but also as an autocrine factor favoring Th1-directed differentiation [19, 20]. Lower concentrations of IFN-γ may be related to the neutralizing anti–interferon-γ autoantibodies, which are increasingly being recognized as a cause of both adult-onset immunodeficiency and increased risk of infections with intracellular pathogens, including *Cryptococcus neoformans*, *Histoplasma capsulatum*, *Talaromyces marneffei*, and disseminated salmonellosis [21]. Neutralizing anti–IFN-γ autoantibodies may neutralize the IFN-γ in the peripheral blood and pleural effusions in non-HIV-infected talaromycosis patients. IL-23 is a central cytokine mediating T helper 17 (Th17) development, including expansion and stabilization. It plays an essential role in extracellular responses to bacteria and fungi, primarily targeting neutrophils [22], an additional table shows this in more detail (see Additional file 1). The roles of these molecules may explain why TMPEs are exudative with predominantly neutrophils, abscess-formation, and high concentration of IL-23 while TPEs had predominantly lymphocytes. In addition, these data may suggest that Th17 cells mediate immunity in TMPEs, while Th1 cells mediate immunity in TPEs, although this will require confirmation.

Pleural fluid levels of ADA, INF-γ, and IL-27 are present in significantly higher concentrations in patients with TPE than those in patients with other types of pleural effusion [11, 23]. This study compared IL-23, IL-27, and IFN-γ concentrations and ADA activity regarding their discriminatory potential to differentiate talaromycosis from tuberculous as well as their ability to help rule in or rule out a diagnosis of TMPE. Using ROC curve analysis, the cutoff value from our study was > 53.07 pg/mL for IL-23, < 129.9 pg/mL for INF-γ, and < 10.9 U/L for ADA may more consider TMPE. Furthermore, the diagnostic accuracy of IL-27 concentrations to discriminate TPE and TMPE was not significant. Thus, higher neutrophil counts and IL-23 concentration and lower ADA activity and IFN-γ concentration may suggest TMPE. Conversely, higher lymphocyte counts, ADA activity, and IFN-γ concentration and lower IL-23 concentration may suggest TPE. Pleural fluid IFN-γ and IL-23 concentrations and ADA activity may be useful diagnostic biomarkers and therefore a rapid, cost-effective, and minimally invasive approach compared to traditional tests to differentiate TMPE from TPE in the pleura.

In the present study, there was a high case-fatality rate of 42.1% (8/19) among patients with TMPEs, due to the long period of misdiagnosis and mistreatment, despite receiving antifungal therapy; in contrast, all the 11 patients who received treatment improved. Drugs such as itraconazole, amphotericin B, fluconazole, and voriconazole can achieve good results. These findings suggest that timely and effective antifungal therapy can improve patient prognosis. The following treatment schema can elicit good results: intravenous amphotericin B deoxycholate 0.6–1.0 mg/kg/day for 2 weeks, followed by oral itraconazole 400 mg/day for 8 to 10 weeks. Other antifungal agents such as fluconazole or voriconazole can also achieve therapeutic efficacy [1, 15]. TMPE can be resorbed in one to 2 weeks following effective antifungal therapy. Upon the onset of dyspnea, chest distress, or other compression symptoms, the effusion can be drained. It is not necessary to use local injections of glucocorticoids, and pleurae adhesion and pachynsis is rare.

Notwithstanding the strengths of this study, it also had limitations. First, the total number of patients enrolled in each group was relatively small, particularly for TMPE, and only three types of cytokines were examined, which is insufficient to comprehensively capture the picture of pathogenesis relating to vascular permeability. Second, although our data showed significant discriminatory ability of IL-23, ADA, and IFN-γ to differentiate between TMPE and TPE, further investigation of specific type of T helper cells that mediate immunity in TMPE was not performed.

## Conclusions

Talaromycosis can infringe on the pleural cavity owing to talaromycosis fungal translocation into the pleural space. TMPEs are usually yellowish and exudative and predominantly composed of neutrophils. Furthermore, higher neutrophil counts and IL-23 concentration and lower ADA activity suggest a diagnosis of talaromycosis. In contrast, higher lymphocyte counts, ADA activity, and IFN-γ concentration suggest a diagnosis of tuberculosis.

## Additional file

Additional file 1: Sources and functions of IL-23 and IFN-γ (DOCX 16 kb)

## Data Availability

The datasets analyzed for the current study are available from the corresponding author upon reasonable request.

## Abbreviations

ADA: Adenosine deaminase
ANOVA: One-way analysis of variance
AUC: Area under the curve
BALF: Bronchoalveolar lavage fluid
CI: Confidence interval
ELISA: Enzyme-linked immunosorbent assay
HIV: Human immunodeficiency virus
IFN-γ: Interferon-gamma
IL: Interleukin
IQR: Interquartile range
LDH: Lactate dehydrogenase
LR: Likelihood ratio
NCC: Nucleated cell count
PAS: Periodic Acid-Schiff
PE: Pleural effusion
SD: Standard deviations
Th: T helper
TMPE: Talaromycosis pleural effusion
TPE: Tuberculosis pleural effusion
