# Supplementary material for: Comparison of pleural effusion features and biomarkers between talaromycosis and tuberculosis in non-human immunodeficiency virus-infected patients
Source: BMC Infect Dis. 2019 Aug 27;19:745. doi: 10.1186/s12879-019-4376-6 (PMC6712812; doi:10.1186/s12879-019-4376-6)
Supplement: Supplementary file 1 — Sources and functions of IL-23 and IFN-γ (DOCX 16 kb) [file 12879_2019_4376_MOESM1_ESM.docx]

Additional file 1 Sources and functions of IL-23 and IFN-γ

| **Cytokine** |  | **Main cell source** | **Cell target** | **Infectious agents** | **Function** |
| --- | --- | --- | --- | --- | --- |
| **IL-23** |  | Monocytes  Dendritic cells | Neutrophils | Extracellular bacteria  Fungi | Th17 expansion and stabilization |
| **IFN-γ** |  | Th1 cells  Natural killer cells | Macrophages  Dendritic cells | Intracellular bacteria  Fungi  Viruses | Cell-mediated immune pathway  Control of intracellular pathogens  Inhibition of Th17 pathway |

IFN, interferon; IL, interleukin; Th, T-helper
